# Hearing Loss in Older Adults: Consistent Determinants Across Two Community‐Based Cohorts in Southern China

**DOI:** 10.1155/jare/4809368

**Published:** 2026-07-07

**Authors:** Dian Zhu, Xutong Zhong, Ruiqiang Li, Xianhai Zeng, Lin Xu, Juanjuan Li

**Affiliations:** ^1^ Department of Epidemiology, School of Public Health, Sun Yat-sen University, Guangzhou, Guangdong, China, sysu.edu.cn; ^2^ Department of Otolaryngology, Shenzhen Longgang Otolaryngology Hospital and Shenzhen Institute of Otolaryngology, Shenzhen, Guangdong, 518172, China; ^3^ SCUT-SLENT Digtal Hearing Healthcare Joint Lab, Shenzhen, Guangdong, 518172, China; ^4^ Department of Applied Health Sciences, University of Birmingham, Birmingham, UK, birmingham.ac.uk; ^5^ School of Public Health, The University of Hong Kong, Hong Kong, China, hku.hk

**Keywords:** community-based study, hearing loss, older adults

## Abstract

**Background:**

Hearing loss (HL) is common in older adults and is associated with substantial functional decline, yet community‐based evidence on its determinants remains limited in China, particularly across different methods of hearing assessment.

**Objectives:**

To investigate associations and predictors of HL among older adults in southern China using audiometric and self‐reported assessments, and to compare patterns across two community‐based cohorts.

**Methods:**

Data were analyzed from 2664 adults aged ≥ 60 years in Shenzhen and 30,518 adults aged ≥ 50 years from the Guangzhou Biobank Cohort Study (GBCS). Moderate‐to‐severe HL was defined as a pure‐tone average (PTA) ≥ 35 dB hearing level in the better‐hearing ear, calculated from air‐conduction thresholds at 500–8000 Hz. HL was assessed using pure‐tone audiometry in Shenzhen and a validated self‐reported measure in GBCS. Multivariable logistic regression estimated adjusted odds ratios (aORs) with 95% confidence intervals (CIs). Extreme gradient boosting with SHAP values was used to assess predictor importance.

**Results:**

Older age, male sex, and lower household income were consistently associated with higher odds of HL in both cohorts. In Shenzhen, metabolic disease (aOR = 1.28, 95% CI: 1.05–1.57) and otitis media (aOR = 2.65, 95% CI: 1.63–4.33) were positively associated with HL, whereas thyroid disease showed an inverse association. In GBCS, alcohol consumption (aOR = 1.28, 95% CI: 1.15–1.43), arthritis (aOR = 1.37, 95% CI: 1.23–1.52), and stroke (aOR = 1.64, 95% CI: 1.06–2.45) were positively associated, while overweight status and nonmanual occupation were inversely associated. Machine‐learning analyses consistently identified age, sex, education, income, and chronic diseases as key predictors.

**Conclusions:**

HL in older adults shows both shared and cohort‐specific associations across assessment methods, highlighting sociodemographic and health‐related disparities. Targeted community‐based screening and prevention strategies are warranted.

## 1. Introduction

Hearing loss (HL) is a major and growing global public health concern as populations age. The World Health Organization estimates that more than 25% of adults aged ≥ 60 years have disabling HL, with prevalence continuing to rise worldwide [1]. Beyond impairing communication and reducing quality of life [2], HL imposes substantial social and economic burdens, with global costs exceeding USD 981 billion in 2019 [3]. Accumulating evidence further links HL to cognitive decline [4–6], depression [7], and frailty [8], compounding multimorbidity in older adults. Despite the availability of effective interventions such as hearing aids, their uptake remains low. In a study, only 29.2% of individuals with HL used hearing aids, with even lower utilization among Black, Hispanic, and low‐income populations [9]. This substantial treatment gap highlights the urgency of improving early identification and prevention strategies in community settings.

While randomized controlled trials (RCTs) and clinic‐based studies have established the efficacy of hearing interventions, their reliance on selected populations or administrative data limits the generalizability of findings to the broader aging community [10–12]. Community‐based, population‐representative data remain scarce in China, and variation in diagnostic approaches further reduces comparability across studies [13, 14]. These gaps highlight the need for large community samples with standardized data collection to better characterize HL and its determinants in real‐world populations.

To address the evidence gap and improve generalizability beyond clinical samples, we examined HL and its associated factors using cross‐sectional data from two independent cohorts of community‐dwelling older adults in southern China. Hearing was assessed using pure‐tone audiometry in Shenzhen and a standardized self‐reported measure in the Guangzhou Biobank Cohort Study (GBCS). By integrating machine‐learning–based prediction with traditional epidemiological analyses, this study aimed to identify robust and potentially modifiable factors of HL.

## 2. Methods

### 2.1. Study Design and Population

This study used data from two independent sources of community‐dwelling older adults in southern China: (1) a routine health screening program for adults aged ≥ 60 years in Longgang District, Shenzhen and (2) the baseline survey of the GBCS. Given differences in hearing assessment methods (audiometry vs. self‐reported measures) and covariate definitions between the two studies, the two study populations were analyzed separately. Study findings are presented comparatively to identify consistent risk factors across populations and measurement approaches.

### 2.2. Shenzhen Sample

Residents aged ≥ 60 years in Longgang District are provided annual health check‐ups at community health centers. Participants were enrolled using consecutive sampling between January and December 2024. Individuals were eligible if they completed pure‐tone audiometry and the risk factor questionnaire. Participants with missing audiometric or PSQI data were excluded. Written informed consent was obtained from all participants. Details of the study have been reported elsewhere [15].

### 2.3. GBCS Sample

Details of GBCS studies have been reported previously [16–18]. Briefly, baseline examinations were conducted from September 2003 to January 2008, including 30,518 participants without self‐reported cardiovascular disease at baseline. GBCS is a collaboration among Guangzhou Twelfth People’s Hospital and the Universities of Hong Kong and Birmingham. Participants were recruited from the Guangzhou Health and Happiness Association for the Respectable Elders (GHHARE), a large community organization for local residents aged ≥ 50 years. Membership requires a nominal monthly fee of 4 RMB and covered approximately 7% of this age group in Guangzhou across all districts.

### 2.4. Outcome Variable

In the Shenzhen sample, trained examiners conducted pure‐tone audiometry in sound‐attenuated environments. Air‐conduction thresholds were measured at 500, 1000, 2000, 4000, and 8000 Hz, consistent with the diagnostic standard pure‐tone audiometry frequency range (0.125–8 kHz) recommended by the ASHA Guidelines for Manual Pure‐Tone Threshold Audiometry [19]. The inclusion of 8000 Hz is further supported by evidence that cochlear damage at frequencies above 8000 Hz precedes threshold shifts at conventional frequencies (250–8000 Hz), rendering high‐frequency measurement particularly sensitive for early detection of age‐related HL [20]. The pure‐tone average (PTA, dB HL) of the better ear was calculated as the mean threshold across these frequencies. Moderate‐to‐severe hearing impairment was defined as PTA ≥ 35 dB HL, while PTA < 35 dB HL was classified as normal/mild impairment. This dichotomous grouping (< 35 vs. ≥ 35 dB HL) served as the primary outcome.

In GBCS, hearing status was self‐assessed at baseline with five response options: *excellent*, *good*, *fair*, *poor*, and *unable to hear*. We classified *poor* and *unable to hear* as HL (coded as 1), and *excellent*/*good*/*fair* as no HL (coded as 0). Although self‐reported hearing has potential for misclassification, research shows that in older adults it exhibits reasonable validity for detecting HL [21].

### 2.5. Risk Factor Data Collection

In the Shenzhen sample, a structured questionnaire and clinical examination were used to collect information on potential HL risk factors. Demographic characteristics included age, sex, educational attainment (primary school or less, middle school, college, or above), annual household income (≤ ¥9999; ¥10,000–49,999; ≥ ¥50,000), and lifelong occupation (manual, nonmanual, and other). Lifestyle factors included smoking status (never vs. ever) and alcohol consumption (never vs. current or former). Medical history included physician‐diagnosed cardiovascular, cerebrovascular, and musculoskeletal diseases, as well as metabolic diseases, defined as one or more of the following physician‐diagnosed conditions: diabetes mellitus or hyperlipidemia. Histories of chronic otitis media and otitis externa were specifically collected due to their relevance to auditory function. Occupational or environmental noise exposure (yes/no) was defined as regular exposure to loud noise without proper hearing protection.

Sleep quality was assessed using the Pittsburgh Sleep Quality Index (PSQI). Seven components (subjective sleep quality, sleep latency, sleep duration, sleep efficiency, sleep disturbances, hypnotic use, and daytime dysfunction) were scored from 0 to 3 and summed to yield a global score (0–21), with higher values indicating poorer sleep quality. Following established recommendations, PSQI > 7 indicated a sleep disorder (coded as 1), while ≤ 7 indicated normal sleep (coded as 0). Component scoring followed the official PSQI guidelines [22].

In GBCS, baseline data were collected through standardized interviews and physical examinations conducted by trained personnel. Demographics included age, sex, and socioeconomic status, defined by education (primary or below, middle school, college, or above), occupation (manual, nonmanual, and others), and annual household income (≤ ¥30,000; > ¥30,000; unknown; 1 USD ≈ 7 RMB). Medical history was based on self‐report and physician diagnosis and included metabolic diseases (diabetes mellitus, hypertension, and hyperlipidemia), cardiovascular diseases (coronary heart disease, stroke, angina, myocardial infarction, or peripheral vascular disease), and other conditions (thyroid disease, fracture history, and arthritis).

Trained personnel measured height and weight to compute body mass index (BMI, kg/m^2^). Blood pressure was measured three times after ≥ 5 min seated rest using an Omron 705CP sphygmomanometer (Omron Corporation, Kyoto, Japan), and the mean of the last two readings was recorded. Hypertension was defined as systolic blood pressure ≥ 140 mmHg or diastolic blood pressure ≥ 90 mmHg. Sleep‐related information included insomnia and hypnotic use > 1 time/week (coded as 1).

### 2.6. Statistical Analysis

Data processing and statistical analysis were performed using Stata 18 and *R* Version 4.3. Multiple imputation by chained equations (MICE) was used to handle missing data by generating five imputed datasets. Continuous variables were summarized as mean ± standard deviation (SD) and compared using *t*‐tests, while categorical variables were expressed as frequency (percentage) and compared using chi‐square tests.

Multivariable logistic regression was used to determine factors independently associated with HL. Multicollinearity was assessed using generalized variance inflation factor (GVIF), with all values < 2.

Additionally, an Extreme Gradient Boosting (XGBoost) model was developed in each sample to assess predictor importance using SHapley Additive exPlanations (SHAP) values. The dataset was split into training (70%) and test (30%) sets using stratified random partitioning. Five‐fold cross‐validation with early stopping (20 rounds) was applied to prevent overfitting. Key hyperparameters included: maximum tree depth = 4, learning rate (eta) = 0.05, gamma = 1, subsample = 0.8, colsample_bytree = 0.8, minimum child weight = 3, L2 regularization (lambda) = 1, and L1 regularization (alpha) = 0.5. To address potential class imbalance in the GBCS sample (negative‐to‐positive ratio approximately 16:1), models with varying scale_pos_weight values (1, 5, 10, and 16.3) were evaluated; as cross‐validation AUC was consistent across all values (range: 0.681–0.682), the unweighted model was retained for its superior training stability. For the GBCS sample, the optimal classification threshold was determined using the Youden index. The primary purpose of the XGBoost analysis was variable importance ranking rather than clinical prediction; performance metrics are reported for methodological transparency.

## 3. Results

### 3.1. Baseline Characteristics

A total of 2664 participants from the Longgang ENT Hospital sample and 30,518 from the GBCS sample were included in the analysis. Table 1 shows that, in the Shenzhen sample, 39.5% (1053 individuals) of participants had moderate or greater HL (PTA ≥ 35 dB). Those with HL were older (mean age 69.4 years, SD 6.4 years) than those without (mean age 66.5 years, SD 5.2 years, *p* < 0.001) and more likely to be male (*p* < 0.001). HL was associated with lower educational level (*p* < 0.001), lower household income (*p* = 0.002), smoking (*p* < 0.001), metabolic disease (*p* = 0.042), and otitis media (*p* < 0.001).

**TABLE 1:** Baseline characteristics of study participants from Shenzhen.

	Total	No or mild hearing loss (PTA < 35 dB) *N* = 1611	Moderate or greater hearing loss (PTA ≥ 35 dB) *N* = 1053	*p* value
Age, years, mean (SD)	2664	66.49 (5.16)	69.41 (6.43)	< 0.001
Sex, *N* (%)				< 0.001
Women	1398 (52.48)	925 (57.42)	473 (44.92)	
Men	1266 (47.52)	686 (42.58)	580 (55.08)	
Obesity status, *N* (%)				0.287
Underweight	28 (1.05)	12 (0.74)	16 (1.52)	
Normal weight	2213 (83.07)	1344 (83.43)	869 (82.53)	
Overweight	373 (14.00)	224 (13.90)	149 (14.15)	
Obesity	50 (1.88)	31 (1.92)	19 (1.80)	
Education, *N* (%)				< 0.001
Primary or below	1231 (46.21)	684 (42.46)	547 (51.95)	
Middle school	1309 (49.14)	840 (52.14)	469 (44.54)	
College or above	124 (4.65)	87 (5.40)	37 (3.51)	
Occupation, *N* (%)				0.290
Manual	2214 (83.11)	1324 (82.18)	890 (84.52)	
Nonmanual	165 (6.19)	105 (6.52)	60 (5.70)	
Others	285 (10.70)	182 (11.30)	103 (9.78)	
Household income, Yuan/year, *N* (%)				0.002
< 10,000	1274 (47.82)	726 (45.07)	548 (52.04)	
10,000–49,999	821 (30.82)	516 (32.03)	305 (28.96)	
≥ 50,000	569 (21.36)	369 (22.91)	200 (18.99)	
Smoking status, *N* (%)				< 0.001
Never	2190 (82.21)	1360 (84.42)	830 (78.82)	
Current/ex‐smoker	474 (17.79)	251 (15.58)	223 (21.18)	
Alcohol use, *N* (%)				0.088
Never	2350 (88.21)	1435 (89.08)	915 (86.89)	
Current/ex‐drinker	314 (11.79)	176 (10.92)	138 (13.11)	
Metabolic disease, *N* (%)				0.042
No	2085 (78.27)	1282 (79.58)	803 (76.26)	
Yes	579 (21.73)	329 (20.42)	250 (23.74)	
Noise exposure, *N* (%)				0.519
No	2633 (98.84)	1594 (98.94)	1039 (98.67)	
Yes	31 (1.16)	17 (1.06)	14 (1.33)	
Cerebrovascular disease, *N* (%)				0.208
No	2621 (98.39)	1589 (98.63)	1032 (98.01)	
Yes	43 (1.61)	22 (1.37)	21 (1.99)	
Thyroid disease, *N* (%)				0.013
No	2642 (99.17)	1592 (98.82)	1050 (99.72)	
Yes	22 (0.83)	19 (1.18)	3 (0.28)	
Musculoskeletal disease, *N* (%)				0.684
No	2580 (96.85)	1562 (96.96)	1018 (96.68)	
Yes	84 (3.15)	49 (3.04)	35 (3.32)	
Otitis media, *N* (%)				< 0.001
No	2584 (97.00)	1578 (97.95)	1006 (95.54)	
Yes	80 (3.00)	33 (2.05)	47 (4.46)	
Otitis externa, *N* (%)				> 0.999
No	2653 (99.59)	1604 (99.57)	1049 (99.62)	
Yes	11 (0.41)	7 (0.43)	4 (0.38)	
Cardiovascular disease, *N* (%)				0.434
No	1530 (57.43)	935 (58.04)	595 (56.51)	
Yes	1134 (42.57)	676 (41.96)	458 (43.49)	
Sleep disorder, *N* (%)				0.389
No	2107 (79.09)	1283 (79.64)	824 (78.25)	
Yes	557 (20.91)	328 (20.36)	229 (21.75)	

Abbreviation: PTA = pure‐tone average (dB).

In the GBCS sample, 5.8% (1763 individuals) of participants reported poor or unable hearing. HL was associated with older age, male sex, lower education, lower household income, underweight status, smoking, alcohol use, arthritis, thyroid disease, hypertension, coronary heart disease, stroke, myocardial infarction, and insomnia (all *p* < 0.05) (Table 2).

**TABLE 2:** Baseline characteristics of study participants in the GBCS cohort.

	Total	Poor or unable to hear *N* = 1763	Normal/mild hearing *N* = 28755	*p* value
Age, years, mean (SD)	30518	61.76 (7.00)	66.51 (7.57)	< 0.001
Sex, *N* (%)				< 0.001
Women	22075 (72.33)	21042 (73.18)	1033 (58.59)	
Men	8443 (27.67)	7713 (26.82)	730 (41.41)	
Obesity status, *N* (%)				0.019
Underweight	1388 (4.55)	1301 (4.52)	87 (4.93)	
Normal weight	14946 (48.97)	14026 (48.78)	920 (52.18)	
Overweight	10998 (36.04)	10417 (36.23)	581 (32.96)	
Obesity	3186 (10.44)	3011 (10.47)	175 (9.93)	
Education, *N* (%)				< 0.001
Primary or below	13100 (42.93)	12181 (42.36)	919 (52.13)	
Middle school	14684 (48.12)	13989 (48.65)	695 (39.42)	
College or above	2734 (8.96)	2585 (8.99)	149 (8.45)	
Occupation, *N* (%)				0.146
Manual	18634 (61.06)	17519 (60.93)	1115 (63.24)	
Nonmanual	7278 (23.85)	6885 (23.94)	393 (22.29)	
Others	4606 (15.09)	4351 (15.13)	255 (14.46)	
Household income, Yuan/year, *N* (%)				< 0.001
< 5000	792 (2.60)	707 (2.46)	85 (4.82)	
5000‐	1846 (6.05)	1716 (5.97)	130 (7.37)	
10000‐	5503 (18.03)	5120 (17.81)	383 (21.72)	
20000‐	7804 (25.57)	7344 (25.54)	460 (26.09)	
30000‐	8237 (26.99)	7820 (27.20)	417 (23.65)	
≥ 50000	6336 (20.76)	6048 (21.03)	288 (16.34)	
Smoking status, *N* (%)				< 0.001
No	24641 (80.74)	23376 (81.29)	1265 (71.75)	
Yes	5877 (19.26)	5379 (18.71)	498 (28.25)	
Alcohol use, *N* (%)				< 0.001
No	20926 (68.57)	19827 (68.95)	1099 (62.34)	
Yes	9592 (31.43)	8928 (31.05)	664 (37.66)	
Frequent hypnotic use (> 1 time/week), *N* (%)				0.089
No	29645 (97.14)	27944 (97.18)	1701 (96.48)	
Yes	873 (2.86)	811 (2.82)	62 (3.52)	
Daytime napping, *N* (%)				0.093
No	10587 (34.69)	10008 (34.80)	579 (32.84)	
Yes	19931 (65.31)	18747 (65.20)	1184 (67.16)	
Arthritis, *N* (%)				< 0.001
No	21087 (69.10)	19954 (69.39)	1133 (64.27)	
Yes	9431 (30.90)	8801 (30.61)	630 (35.73)	
Thyroid disease, *N* (%)				0.002
No	27885 (91.37)	26238 (91.25)	1647 (93.42)	
Yes	2633 (8.63)	2517 (8.75)	116 (6.58)	
Fracture history, *N* (%)				0.428
No	25974 (85.11)	24485 (85.15)	1489 (84.46)	
Yes	4544 (14.89)	4270 (14.85)	274 (15.54)	
Diabetes mellitus, *N* (%)				0.633
No	28099 (92.07)	26481 (92.09)	1618 (91.78)	
Yes	2419 (7.93)	2274 (7.91)	145 (8.22)	
Hypertension, *N* (%)				< 0.001
No	20889 (68.45)	19775 (68.77)	1114 (63.19)	
Yes	9629 (31.55)	8980 (31.23)	649 (36.81)	
Hyperlipidemia, *N* (%)				0.277
No	27323 (89.53)	25731 (89.48)	1592 (90.30)	
Yes	3195 (10.47)	3024 (10.52)	171 (9.70)	
Cardiovascular disease, N (%)				0.160
No	17885 (58.60)	16880 (58.70)	1005 (57.01)	
Yes	12633 (41.40)	11875 (41.30)	758 (42.99)	
Coronary heart disease, *N* (%)				0.004
No	29472 (96.57)	27791 (96.65)	1681 (95.35)	
Yes	1046 (3.43)	964 (3.35)	82 (4.65)	
Stroke, *N* (%)				< 0.001
No	30292 (99.26)	28557 (99.31)	1735 (98.41)	
Yes	226 (0.74)	198 (0.69)	28 (1.59)	
Angina pectoris, *N* (%)				0.062
No	30244 (99.10)	28504 (99.13)	1740 (98.70)	
Yes	274 (0.90)	251 (0.87)	23 (1.30)	
Myocardial infarction, *N* (%)				0.023
No	30407 (99.64)	28656 (99.66)	1751 (99.32)	
Yes	111 (0.36)	99 (0.34)	12 (0.68)	
Peripheral vascular disease, *N* (%)				0.101
No	30475 (99.86)	28717 (99.87)	1758 (99.72)	
Yes	43 (0.14)	38 (0.13)	5 (0.28)	
Insomnia, *N* (%)				0.012
No	25300 (82.90)	23877 (83.04)	1423 (80.71)	
Yes	5218 (17.10)	4878 (16.96)	340 (19.29)	

### 3.2. Association Between Participant Characteristics and HL

Table 3 shows that in the Shenzhen sample, after mutual adjustment for all variables in the model, older age (aOR = 1.09, 95% CI: 1.07–1.11), male sex (aOR = 1.84, 95% CI: 1.52–2.24), metabolic disease (aOR = 1.28, 95% CI: 1.05–1.57), and otitis media (aOR = 2.65, 95% CI: 1.63–4.33) were independently and positively associated with HL. Higher educational level (aOR ranging from 0.58 to 0.75, *p* < 0.03), higher household income (aOR ranging from 0.72 to 0.82, *p* < 0.05), and thyroid disease (aOR = 0.26, 95% CI: 0.06–0.81) were inversely associated with HL.

**TABLE 3:** Univariable and multivariable logistic regression analysis of hearing loss among participants from Shenzhen.

Variable	Crude OR (95% CI)	Crude *p* value	Adjusted OR (95% CI)^†^	Adjusted *p* value
Age, years	1.09 (1.08–1.11)	< 0.001	1.09 (1.07–1.11)	< 0.001
Sex				
Women	Reference		Reference	
Men	1.65 (1.41–1.93)	< 0.001	1.84 (1.52–2.24)	< 0.001
Obesity status				
Underweight	2.06 (0.98–4.48)	0.060	1.72 (0.78–3.87)	0.180
Normal weight	Reference		Reference	
Overweight	1.03 (0.82–1.29)	0.804	1.05 (0.83–1.34)	0.672
Obesity	0.95 (0.52–1.67)	0.856	1.17 (0.63–2.13)	0.611
Education				
Primary or below	Reference		Reference	
Middle school	0.70 (0.60–0.82)	< 0.001	0.75 (0.62–0.89)	0.001
College or above	0.53 (0.35–0.79)	0.002	0.58 (0.36–0.93)	0.027
Occupation				
Manual	Reference		Reference	
Nonmanual	0.85 (0.61–1.18)	0.332	0.88 (0.60–1.31)	0.542
Others	0.84 (0.65–1.09)	0.188	0.86 (0.63–1.16)	0.330
Household income, Yuan/year				
< 10,000	Reference		Reference	
10,000–49,999	0.78 (0.65–0.94)	0.008	0.82 (0.67–0.99)	0.044
≥ 50,000	0.72 (0.58–0.88)	0.002	0.72 (0.56–0.92)	0.009
Smoking status				
Never	Reference		Reference	
Current/ex‐smoker	1.46 (1.19–1.78)	< 0.001	1.09 (0.85–1.40)	0.482
Alcohol use				
Never	Reference		Reference	
Current/ex‐drinker	1.23 (0.97–1.56)	0.088	1.05 (0.80–1.38)	0.728
Metabolic disease				
No	Reference		Reference	
Yes	1.21 (1.01–1.46)	0.042	1.28 (1.05–1.57)	0.015
Noise exposure				
No	Reference		Reference	
Yes	1.26 (0.61–2.57)	0.520	1.27 (0.59–2.71)	0.540
Cerebrovascular disease				
No	Reference		Reference	
Yes	1.47 (0.80–2.69)	0.211	1.17 (0.61–2.22)	0.632
Thyroid disease				
No	Reference		Reference	
Yes	0.24 (0.06–0.70)	0.022	0.26 (0.06–0.81)	0.039
Musculoskeletal disease				
No	Reference		Reference	
Yes	1.10 (0.70–1.70)	0.684	1.24 (0.76–1.99)	0.384
Otitis media				
No	Reference		Reference	
Yes	2.23 (1.43–3.54)	< 0.001	2.65 (1.63–4.33)	< 0.001
Otitis externa				
No	Reference		Reference	
Yes	0.87 (0.23–2.90)	0.830	0.78 (0.19–2.79)	0.715
Cardiovascular disease				
No	Reference		Reference	
Yes	1.06 (0.91–1.25)	0.434	0.95 (0.80–1.13)	0.560
Sleep disorder				
No	Reference		Reference	
Yes	1.09 (0.90–1.31)	0.389	1.04 (0.85–1.28)	0.679

^†^Adjusted for age, sex, educational level, occupation, household income, smoking, alcohol consumption, obesity status, comorbidities (metabolic disease, cardiovascular disease, thyroid disease, and musculoskeletal disease), noise exposure, otitis media, otitis externa, and sleep quality.

In the GBCS sample, after mutual adjustment for all variables in the model, older age (aOR = 1.09, 95% CI: 1.08–1.10) and male sex (aOR = 1.64, 95% CI: 1.43–1.88) were strongly and positively associated with HL. Alcohol consumption (aOR = 1.28, 95% CI: 1.15–1.43), arthritis (aOR = 1.37, 95% CI: 1.23–1.52), and stroke (aOR = 1.64, 95% CI: 1.06–2.45) also showed significant positive associations. In contrast, nonmanual occupation (aOR = 0.77, 95% CI: 0.66–0.88), overweight status (aOR = 0.86, 95% CI: 0.77–0.96), and higher household income (aOR ranging from 0.63 to 0.70, *p* < 0.01) were inversely associated with HL (Table 4).

**TABLE 4:** Univariable and multivariable logistic regression analysis of hearing loss in the GBCS cohort.

Variable	Crude OR (95% CI)	Crude *p* value	Adjusted OR (95% CI)^†^	Adjusted *p* value
Age, years	1.09 (1.09–1.10)	< 0.001	1.09 (1.08–1.10)	< 0.001
Sex				
Women	Reference		Reference	
Men	1.93 (1.75–2.13)	< 0.001	1.64 (1.43–1.88)	< 0.001
Obesity status				
Underweight	1.02 (0.81–1.27)	0.868	0.86 (0.68–1.08)	0.207
Normal weight	Reference		Reference	
Overweight	0.85 (0.76–0.95)	0.003	0.86 (0.77–0.96)	0.009
Obesity	0.89 (0.75–1.04)	0.154	0.92 (0.77–1.09)	0.335
Education				
Primary or below	Reference		Reference	
Middle school	0.66 (0.59–0.73)	< 0.001	0.95 (0.84–1.07)	0.403
College or above	0.76 (0.64–0.91)	0.003	0.84 (0.68–1.04)	0.108
Occupation				
Manual	Reference		Reference	
Nonmanual	0.90 (0.80–1.01)	0.071	0.77 (0.66–0.88)	< 0.001
Others	0.92 (0.80–1.06)	0.248	1.01 (0.87–1.17)	0.918
Household income, Yuan/year				
< 5000	Reference		Reference	
5000‐	0.63 (0.47–0.84)	0.002	0.66 (0.50–0.89)	0.006
10000‐	0.62 (0.49–0.80)	< 0.001	0.70 (0.55–0.91)	0.006
20000‐	0.52 (0.41–0.67)	< 0.001	0.67 (0.52–0.87)	0.002
30000‐	0.44 (0.35–0.57)	< 0.001	0.68 (0.53–0.89)	0.004
≥ 50000	0.40 (0.31–0.51)	< 0.001	0.63 (0.48–0.83)	< 0.001
Smoking status				
No	Reference		Reference	
Yes	1.71 (1.53–1.90)	< 0.001	0.94 (0.82–1.08)	0.403
Alcohol use				
No	Reference		Reference	
Yes	1.34 (1.21–1.48)	< 0.001	1.28 (1.15–1.43)	< 0.001
Frequent hypnotic use (> 1 time/week)				
No	Reference		Reference	
Yes	1.26 (0.96–1.62)	0.089	1.15 (0.86–1.52)	0.328
Daytime napping				
No	Reference		Reference	
Yes	1.09 (0.99–1.21)	0.093	0.98 (0.88–1.09)	0.715
Arthritis				
No	Reference		Reference	
Yes	1.26 (1.14–1.39)	< 0.001	1.37 (1.23–1.52)	< 0.001
Thyroid disease				
No	Reference		Reference	
Yes	0.73 (0.60–0.89)	0.002	0.88 (0.72–1.06)	0.190
Fracture history				
No	Reference		Reference	
Yes	1.06 (0.92–1.20)	0.428	1.01 (0.88–1.16)	0.871
Diabetes mellitus				
No	Reference		Reference	
Yes	1.04 (0.87–1.24)	0.633	0.97 (0.81–1.16)	0.738
Hypertension				
No	Reference		Reference	
Yes	1.28 (1.16–1.42)	< 0.001	0.97 (0.87–1.08)	0.538
Hyperlipidemia				
No	Reference		Reference	
Yes	0.91 (0.77–1.07)	0.277	0.95 (0.79–1.13)	0.552
Cardiovascular disease				
No	Reference		Reference	
Yes	1.07 (0.97–1.18)	0.160	0.95 (0.84–1.07)	0.399
Coronary heart disease				
No	Reference		Reference	
Yes	1.41 (1.11–1.76)	0.004	1.06 (0.83–1.35)	0.629
Stroke				
No	Reference		Reference	
Yes	2.33 (1.53–3.41)	< 0.001	1.64 (1.06–2.45)	0.020
Angina pectoris				
No	Reference		Reference	
Yes	1.50 (0.95–2.25)	0.064	1.24 (0.77–1.90)	0.346
Myocardial infarction				
No	Reference		Reference	
Yes	1.98 (1.03–3.47)	0.026	1.28 (0.66–2.29)	0.434
Peripheral vascular disease				
No	Reference		Reference	
Yes	2.15 (0.74–4.98)	0.108	1.52 (0.51–3.68)	0.402
Insomnia				
No	Reference		Reference	
Yes	1.17 (1.03–1.32)	0.012	1.12 (0.98–1.27)	0.105

^†^Adjusted for age, sex, education, occupation, household income, physical exposure, insomnia, hypnotic use, daytime napping, arthritis, thyroid disease, fracture history, diabetes mellitus, hypertension, hyperlipidemia, cardiovascular disease, coronary heart disease, stroke, angina, myocardial infarction, peripheral vascular disease, smoking, alcohol use, and BMI, as appropriate.

### 3.3. XGBoost Model and Feature Importance

In the Shenzhen sample, the XGBoost model achieved an AUC of 0.694 on the held‐out test set (training AUC: 0.710; accuracy: 67.2%; sensitivity: 40.3%; specificity: 84.7%). In the GBCS sample, the model achieved an AUC of 0.695 (training AUC: 0.708; accuracy: 66.6%; sensitivity: 65.3%; specificity: 66.7%), with an optimal classification threshold of 0.108. As the primary aim was variable importance ranking, these metrics are reported for transparency rather than predictive utility. ROC curves for both models are presented in Supporting Figures 1 and 2.

**FIGURE 1:**
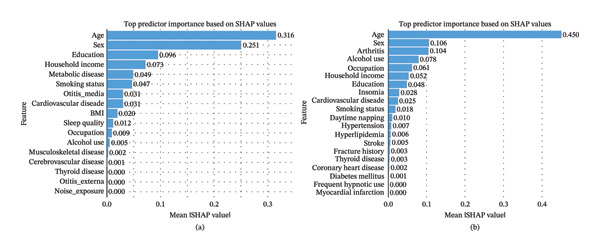
These figures depict top predictor importance based on SHAP values in the XGBoost model. Subpanels (a) and (b) correspond to the Shenzhen cohort and the GBCS cohort, respectively. The horizontal axis shows the mean absolute SHAP values, and the vertical axis lists the predictors included in the model. Bars represent the relative contribution of each predictor to the model’s output as estimated by SHAP.

SHAP value analysis identified age, sex, education, household income, and smoking status as the most influential predictors across both samples. In the GBCS sample, age showed the highest mean |SHAP| value (0.191), followed by sex (0.042), education (0.025), arthritis (0.022), alcohol use (0.015), household income (0.014), and smoking status (0.014). In the Shenzhen sample, age, sex, and education were also leading predictors, while metabolic disease contributed more prominently compared with the GBCS sample (Figure 1). Multicollinearity testing showed that all predictors had adjusted GVIFs below 2, indicating no significant multicollinearity (Supporting Tables 1 and 2).

### 3.4. Sensitivity Analysis

The sensitivity analysis restricted to GBCS participants aged ≥ 60 years (*n* = 17,120; HL prevalence: 8.0%) yielded a similar pattern of predictor importance in the XGBoost model. Age remained the dominant predictor (mean |SHAP| = 0.082), followed by sex (0.037), alcohol use (0.011), and household income (0.006), broadly consistent with the findings from the full GBCS sample (Supporting Figure 3).

## 4. Discussion

In this study, we analyzed two population‐based samples to examine factors associated with HL in older adults. In the Shenzhen sample, metabolic disease and otitis media showed stronger positive associations with HL, whereas in the GBCS, alcohol consumption and arthritis were more prominently associated. In contrast, thyroid disease in the Shenzhen sample and overweight status and nonmanual occupation in the GBCS sample were inversely associated with HL. Despite these cohort‐specific patterns, several associations showed broadly similar directional trends across the two cohorts. Although HL was assessed using different approaches—pure‐tone audiometry in the Shenzhen sample and self‐reported hearing status in the GBCS sample—self‐reported measures may be influenced by educational attainment, cognitive status, health awareness, and sociocultural perceptions of hearing difficulties, potentially introducing reporting bias and limiting direct comparability between cohorts. Nevertheless, associations for older age, male sex, lower household income, and smoking remained consistent across both cohorts despite differences in hearing ascertainment, suggesting that these findings may not be entirely attributable to measurement‐specific bias and may instead reflect broader population‐level patterns of HL in older adults.

Our findings align with previous reports. Results are consistent with previous studies identifying age, male sex [23, 24], and lower income as major risk factors for age‐related HL [25, 26]. The association between metabolic disease and HL observed in the Shenzhen sample also accords with findings from a systematic review by Rim et al. [27] and a cross‐sectional study in the Democratic Republic of Congo [28]. Furthermore, smoking emerged as a significant factor associated with HL in both samples, consistent with previous epidemiological studies suggesting that tobacco exposure may contribute to age‐related auditory decline and the burden of hearing impairment [29–32]. In addition, alcohol consumption—representing an adverse lifestyle factor—was significantly associated with HL, in accordance with evidence from an Australian cohort study [33], a cross‐sectional study in England [26], and a Japanese cross‐sectional study [34]. Taken together, these findings further support the multifactorial nature of age‐related HL and highlight the potential contribution of metabolic conditions and adverse lifestyle factors, particularly smoking and alcohol consumption, to auditory decline in older adults.

A notable and clinically relevant finding in our data is the independent association between arthritis and HL in the GBCS sample. Emerging evidence suggests an increased prevalence of hearing impairment in patients with inflammatory arthritis, particularly rheumatoid arthritis [25, 35]. Several mechanisms could explain this association. First, systemic inflammation characteristic of inflammatory arthritis may contribute to cochlear microvascular dysfunction, immune‐mediated injury, and neuroinflammation in the inner ear, with cytokines such as TNF‐α and IL‐1β implicated in cochlear degeneration [36–38]. Autoimmune inner‐ear processes (disease‐specific autoimmunity or cross‐reactive antibodies) may also precipitate sensorineural injury in susceptible individuals [39–41]. Additionally, comorbid vascular disease or shared cardiometabolic risk factors common in people with arthritis may induce ischemic injury to the cochlea via microvascular compromise [42–46]. Prolonged exposure to analgesics and anti‐inflammatory agents, such as regular NSAID or high‐dose salicylate use, has been associated with increased risk of hearing impairment and may exacerbate cochlear vulnerability [47–49]. These pathways—chronic low‐grade inflammation, immune dysregulation, microvascular insufficiency, and drug‐related ototoxicity—overlap with biological processes implicated in age‐related auditory decline, including inflammaging [50] and cochlear microvascular deterioration [51, 52], and may therefore accelerate HL in older adults [53, 54].

This study has several strengths. First, both samples were derived from population‐based cohorts, enabling the examination of factors associated with HL in populations that more accurately reflect real‐world aging communities and helping address the current evidence base largely shaped by clinic‐based studies. Second, analysis of two independent community samples enhanced the robustness and generalizability of the findings. Third, the application of both traditional multivariable logistic regression and machine‐learning approaches produced broadly consistent results, strengthening confidence in the observed associations.

This study also has limitations. First, the analysis was based on cross‐sectional data from two cities in southern China, which limits generalizability and precludes causal inference. Second, we acknowledge that inclusion of the 8000 Hz threshold may yield higher prevalence estimates compared with studies using the conventional four‐frequency PTA (0.5, 1, 2, and 4 kHz). However, high‐frequency hearing assessment is considered more sensitive for detecting early cochlear damage and may facilitate earlier identification of age‐related HL. Third, the two cohorts differed in age eligibility criteria (≥ 60 years in Shenzhen vs. ≥ 50 years in GBCS), and this difference may partially account for the substantially lower HL prevalence observed in GBCS (5.8% vs. 39.5%), which should be considered when interpreting cross‐cohort comparisons of prevalence estimates. However, the sensitivity analysis suggests that the observed cross‐cohort consistency in key determinants is robust to differences in age structure. At last, several factors potentially associated with HL were not available in the present datasets, including hearing aid use, ototoxic medication exposure, detailed occupational noise exposure history, and cognitive status. These factors may influence both hearing perception and hearing‐related behaviors and could therefore contribute to residual confounding or biased effect estimates. Further studies with more comprehensive assessments are needed to clarify these associations.

## 5. Conclusion

In two large community‐based cohorts of older adults in southern China, we identified both shared and cohort‐specific associations with HL. Older age, male sex, and lower socioeconomic status showed broadly consistent associations across cohorts, while metabolic conditions and adverse lifestyle factors, particularly smoking and alcohol consumption, may also contribute to HL in aging populations. These findings highlight persistent disparities in hearing health and support the need for improved early identification and targeted prevention strategies in community settings.

## Funding

No funding was received for this manuscript.

## Conflicts of Interest

The authors declare no conflicts of interest.

## Supporting Information

Additional supporting information can be found online in the Supporting Information section.

## Supporting information


**Supporting Information** Supporting information is available online and includes additional tables and figures supporting the findings of this study. Specifically, the supporting section provides multicollinearity diagnostics (Supporting Tables 1–2), XGBoost model performance metrics and sensitivity analyses (Supporting Tables 3–4), receiver operating characteristic curves for the Shenzhen and GBCS XGBoost models (Supporting Figures 1–2), and SHAP importance rankings for the GBCS subsample aged ≥ 60 years (Supporting Figure 3).

## Data Availability

The data that support the findings of this study are available on request from the corresponding author. The data are not publicly available due to privacy or ethical restrictions.
