# Supplementary material for: Hearing Loss in Older Adults: Consistent Determinants Across Two Community‐Based Cohorts in Southern China
Source: J Aging Res. 2026 Jul 7;2026:4809368. doi: 10.1155/jare/4809368 (PMC13338764; doi:10.1155/jare/4809368)

**Supplementary material**

**Supplementary Table 1.** Results of Multicollinearity test of predictor variables for participants from Shenzhen

| Variable | GVIF | Df | Adjusted GVIF |
| --- | --- | --- | --- |
| Age | 1.071 | 1 | 1.035 |
| Obesity status | 1.050 | 3 | 1.008 |
| Household income | 1.434 | 2 | 1.094 |
| Occupation | 1.582 | 2 | 1.122 |
| Education | 1.417 | 2 | 1.091 |
| Sex | 1.424 | 1 | 1.193 |
| Alcohol use | 1.227 | 1 | 1.108 |
| Metabolic disease | 1.051 | 1 | 1.025 |
| Cardiovascular disease | 1.067 | 1 | 1.033 |
| Noise exposure | 1.013 | 1 | 1.007 |
| Cerebrovascular disease | 1.039 | 1 | 1.019 |
| Thyroid disease | 1.018 | 1 | 1.009 |
| Musculoskeletal disease | 1.048 | 1 | 1.024 |
| Otitis media | 1.020 | 1 | 1.010 |
| Otitis externa | 1.014 | 1 | 1.007 |
| Smoking status | 1.422 | 1 | 1.192 |
| Sleep Quality | 1.041 | 1 | 1.020 |

VIF = variance inflation factor. GVIF = generalized variance inflation factor. Values above 5 (or 10) may indicate multicollinearity. GVIF^(1/(2*Df)) adjusts GVIF for the number of degrees of freedom.

**Supplementary Table 2.** Multicollinearity test of predictor variables in the GBCS cohort

| Variable | GVIF | Df | Adjusted GVIF |
| --- | --- | --- | --- |
| Age | 1.264 | 1 | 1.124 |
| Sex | 1.898 | 1 | 1.378 |
| BMI | 1.085 | 3 | 1.014 |
| Education | 1.642 | 2 | 1.132 |
| Occupation | 1.421 | 2 | 1.092 |
| Household income | 1.253 | 5 | 1.023 |
| Insomnia | 1.123 | 1 | 1.060 |
| Frequent hypnotic use (>1 time/week) | 1.122 | 1 | 1.059 |
| Daytime napping | 1.035 | 1 | 1.017 |
| Arthritis | 1.042 | 1 | 1.021 |
| Thyroid disease | 1.026 | 1 | 1.013 |
| Fracture history | 1.007 | 1 | 1.004 |
| Diabetes mellitus | 1.033 | 1 | 1.016 |
| Hypertension | 1.149 | 1 | 1.072 |
| Hyperlipidaemia | 1.201 | 1 | 1.096 |
| Cardiovascular disease | 1.428 | 1 | 1.195 |
| Coronary heart disease | 1.093 | 1 | 1.046 |
| Stroke | 1.031 | 1 | 1.015 |
| Angina pectoris | 1.037 | 1 | 1.018 |
| Myocardial infarction | 1.018 | 1 | 1.009 |
| Peripheral vascular disease | 1.007 | 1 | 1.004 |
| Smoking status | 1.658 | 1 | 1.287 |
| Alcohol use | 1.125 | 1 | 1.061 |

VIF = variance inflation factor. GVIF = generalized variance inflation factor. Values above 5 (or 10) may indicate multicollinearity. GVIF^(1/(2*Df)) adjusts GVIF for the number of degrees of freedom.

**Supplementary Table 3.** XGBoost model performance metrics — Shenzhen and GBCS samples

| Metric | Shenzhen | GBCS (full sample) |
| --- | --- | --- |
| Training AUC | 0.710 | 0.708 |
| Test AUC | 0.694 | 0.695 |
| Accuracy | 67.2% | 66.6% |
| Sensitivity | 40.3% | 65.3% |
| Specificity | 84.7% | 66.7% |
| F1 Score | 0.492 | 0.184 |
| Classification threshold | 0.500 | 0.108 |
| Training/test split | 70/30 | 70/30 |
| Cross-validation | 5-fold | 5-fold |

**Supplementary Table 4.** Sensitivity Analysis of scale_pos_weight — GBCS Sample

| scale_pos_weight | Optimal boosting rounds | Cross-validation AUC |
| --- | --- | --- |
| 1 | 43 | 0.682 |
| 5 | 9 | 0.682 |
| 10 | 8 | 0.682 |
| 16.3 (ratio-based) | 10 | 0.681 |

**Supplementary Figure 1.** ROC curve — Shenzhen sample

**
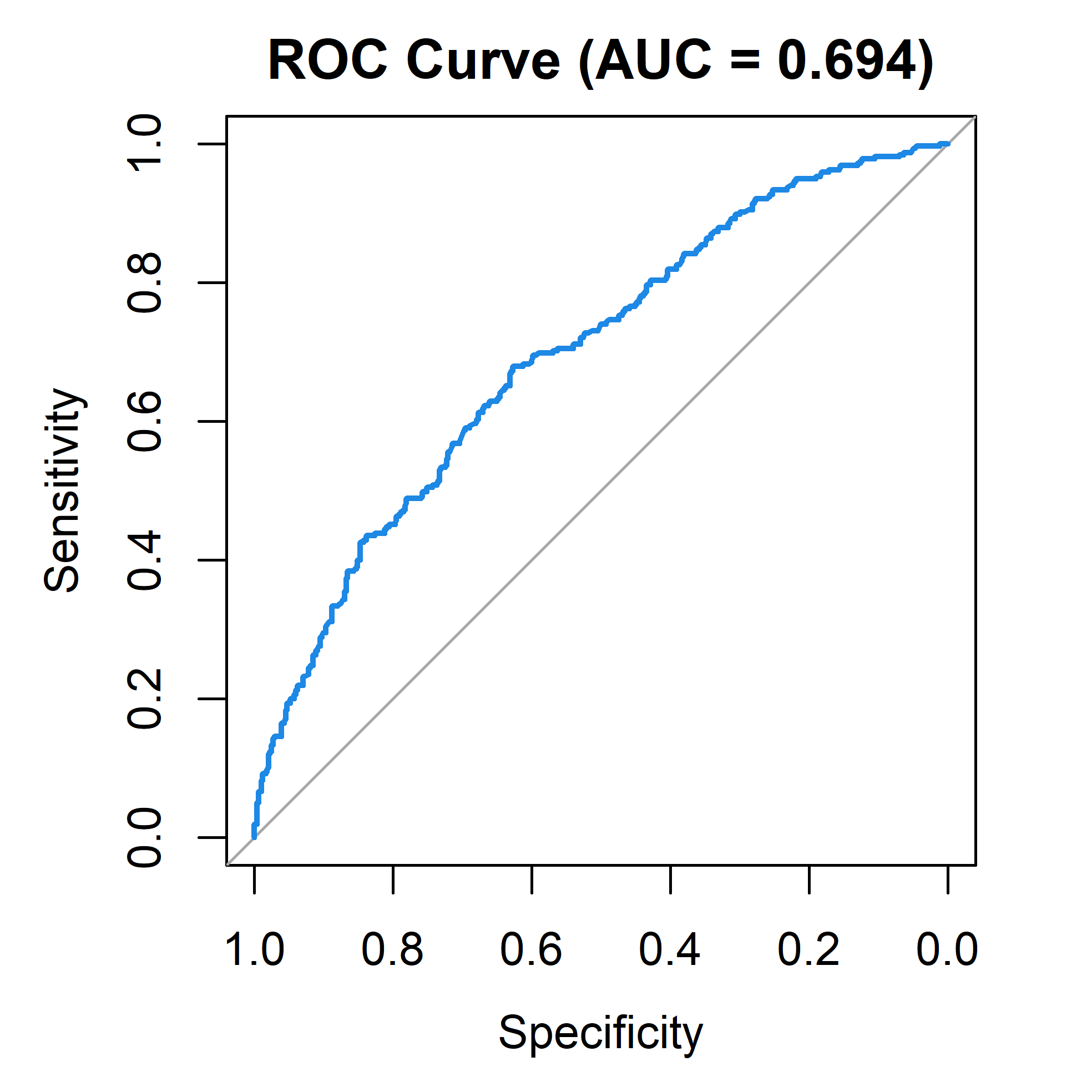
**

**Supplementary Figure 2.** ROC curve — GBCS sample


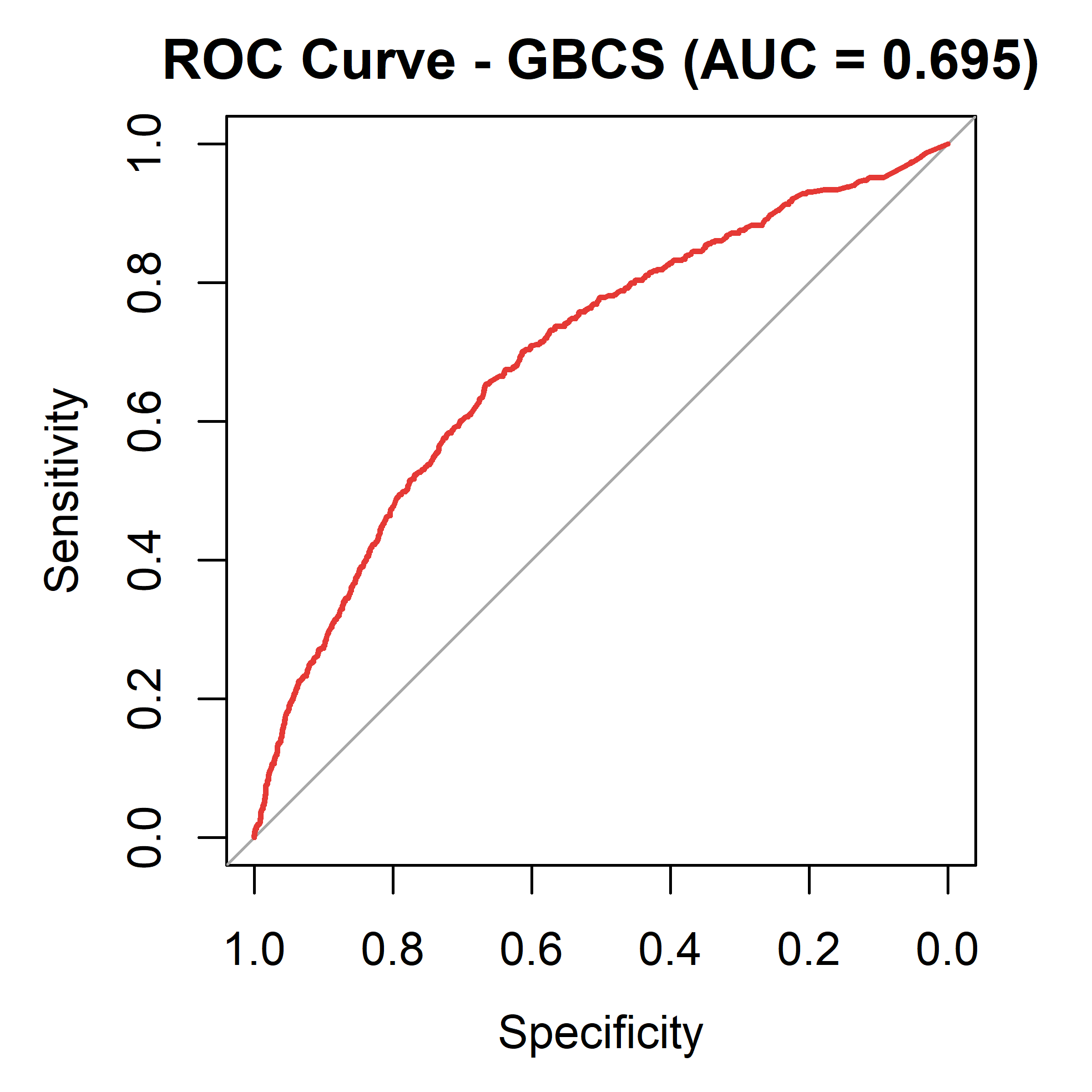


**Supplementary Figure 3.** SHAP importance rankings — GBCS subsample aged ≥60 years


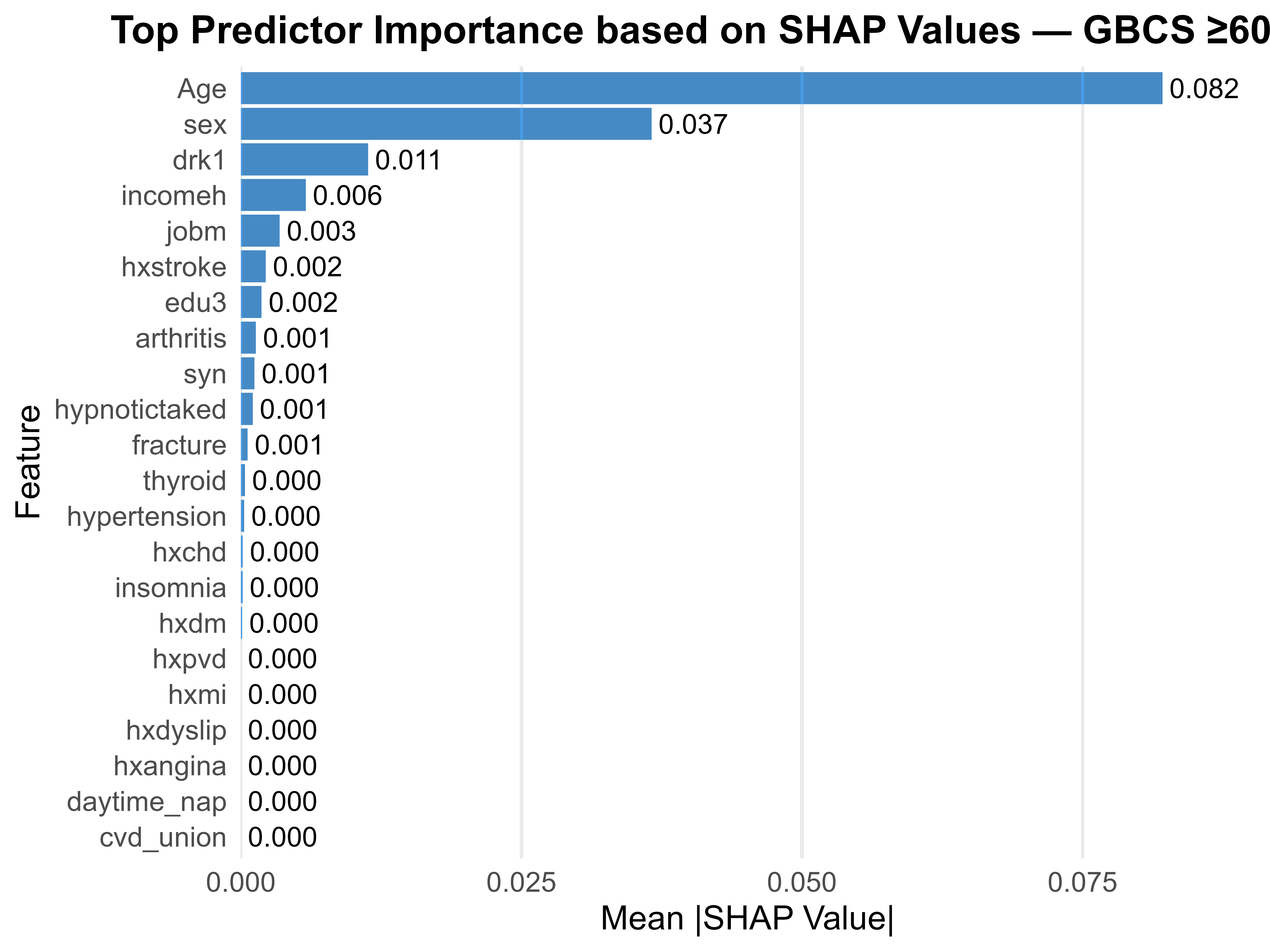

Supplement: Supplementary file 1 — Supporting Information Supporting information is available online and includes additional tables and figures supporting the findings of this study. Specifically, the supporting section provides multicollinearity diagnostics (Supporting Tables 1–2), XGBoost model performance metrics and sensitivity analyses (Supporting Tables 3–4), receiver operating characteristic curves for the Shenzhen and GBCS XGBoost models (Supporting Figures 1–2), and SHAP importance rankings for the GBCS subsample aged ≥ 60 years (Supporting Figure 3). [file JARE-2026-4809368-s001.docx]
